# Correlation of *SHOX2 *Gene Amplification and DNA Methylation in Lung Cancer Tumors

**DOI:** 10.1186/1471-2407-11-102

**Published:** 2011-03-22

**Authors:** Katja U Schneider, Dimo Dietrich, Michael Fleischhacker, Gunda Leschber, Johannes Merk, Frank Schäper, Henk R Stapert, Erik R Vossenaar, Sabine Weickmann, Volker Liebenberg, Christoph Kneip, Anke Seegebarth, Fikret Erdogan, Gudrun Rappold, Bernd Schmidt

**Affiliations:** 1 University Hospital Heidelberg, Institute of Human Genetics, Germany; 2 Epigenomics AG, Berlin, Germany; 3 Medizinische Klinik m.S. Onkologie Hämatologie, Charité-Universitätsmedizin, Berlin, Germany; 4 ELK Berlin Chest Hospital, Germany; 5 Biocartis B.V., Eindhoven, the Netherlands; 6 Medizinische Klinik m.S. Infektiologie und Pneumologie, Pneumologie, Charité-Universitätsmedizin, Berlin, Germany; 7 Metanomics Health GmbH, Berlin, Germany; 8 Theracode GmbH, Mainz, Germany; 9 Bavarian Nordic GmbH, Berlin, Germany; 10 ATLAS Biolabs GmbH, Berlin, Germany; 11 Universitätsklinik und Poliklinik für Innere Medizin I, Universitätsklinikum Halle (Saale), Halle, Germany

## Abstract

**Background:**

DNA methylation in the *SHOX2 *locus was previously used to reliably detect lung cancer in a group of critical controls, including 'cytologically negative' samples with no visible tumor cell content, at a high specificity based on the analysis of bronchial lavage samples. This study aimed to investigate, if the methylation correlates with *SHOX2 *gene expression and/or copy number alterations. An amplification of the *SHOX2 *gene locus together with the observed tumor-specific hypermethylation might explain the good performance of this marker in bronchial lavage samples.

**Methods:**

*SHOX2 *expression, gene copy number and DNA methylation were determined in lung tumor tissues and matched morphologically normal adjacent tissues (NAT) from 55 lung cancer patients. Quantitative HeavyMethyl (HM) real-time PCR was used to detect *SHOX2 *DNA methylation levels. *SHOX2 *expression was assayed with quantitative real-time PCR, and copy numbers alterations were measured with conventional real-time PCR and array CGH.

**Results:**

A hypermethylation of the *SHOX2 *locus in tumor tissue as compared to the matched NAT from the same patient was detected in 96% of tumors from a group of 55 lung cancer patients. This correlated highly significantly with the frequent occurrence of copy number amplification (p < 0.0001), while the expression of the *SHOX2 *gene showed no difference.

**Conclusions:**

Frequent gene amplification correlated with hypermethylation of the *SHOX2 *gene locus. This concerted effect qualifies *SHOX2 *DNA methylation as a biomarker for lung cancer diagnosis, especially when sensitive detection is needed, i.e. in bronchial lavage or blood samples.

## Background

Lung cancer represents the second most common cancer in both men and women and accounts for about 15% of all cancer diagnoses [1]. In the absence of screening, lung cancer patients either exhibit symptoms or are accidentally diagnosed by clinical imaging performed for other indications. Patients suspected of having malignant lung disease usually undergo clinical investigation (workup) including CT-scanning of the thorax and bronchoscopy. The latter is preferentially applied to confirm a lung neoplasm by pathological assessment of tissue or a cytological specimen obtained during the procedure. A final diagnosis after the first bronchoscopy fails in about half of these patients [2], which requires additional invasive diagnostic procedures. Even when signs, symptoms and radiological findings are such that the clinical diagnosis of malignant lung disease appears obvious, it often takes considerable effort and invasive procedures to obtain tissue material suitable for definitively confirming the presence of malignant disease.

Ambiguous results following bronchoscopy are frequent (i.e. the presence of a malignancy cannot be confirmed), e.g. because the tumor is not visible endoscopically and cells obtained by brushing or aspiration do not allow the pathologist to confirm or exclude malignancy.

Biomarkers exhibit great potential for improving the management of cancer in clinical routine. So far, several biomarkers from genetic, proteomic and epigenetic approaches are available for clinical research purposes [3-5]. The use of DNA methylation as a biomarker is an emerging field that provides potential for advancing the clinical process of cancer diagnosis [6-10]. Methylation of DNA is an important epigenetic process involved in fundamental biological events such as development and cell differentiation [for review: [11]]. Aberrant DNA methylation has been reported to play a major role in carcinogenesis [for review: [12]], suggesting that DNA methylation analysis may be a valuable source for cancer biomarkers [13]. Recently, *SHOX2 *DNA methylation was shown to be a useful biomarker for detecting cancer patients at high specificity and sensitivity in a group of critical controls based on the analysis of bronchial lavage samples [14]. Interestingly, samples that were classified as 'cytologically negative' or 'inconclusive' due to no (or too few) visible tumor cell content could be identified as cancer-positive, based on their *SHOX2 *DNA methylation level.

*SHOX2 *has been identified as highly homologous to the short stature homeobox gene *SHOX*. Both homeodomain-transcription factors are involved in skeletogenesis, and the murine *Shox2 *gene has been shown to play a pivotal role during heart development [15-18].

Genomic gain of chromosome 3q involving the *SHOX2 *gene has been recognized as one of the most prevalent and significant chromosomal rearrangements in lung cancer [19-23]. The positive performance of *SHOX2 *DNA methylation as a biomarker in cytologically negative bronchial lavage samples might be due to the concerted effects of locus amplification and DNA methylation of *SHOX2 *in tumor cells. As a result, an increase of *SHOX2 *DNA copies in tumor cells compared to normal cells also increases the *SHOX2 *DNA methylation level in a mixed sample of tumor and normal cells as compared to the methylation level of normal cells alone. In this study the DNA methylation, gene expression and gene amplification of *SHOX2 *in matched tumor and normal adjacent tissues (NAT) from 55 lung cancer patients were investigated.

## Methods

### Patients

The study was comprised of matched morphologically normal lung tissues and tumor tissues from 55 lung cancer patients who underwent surgery. Surgical samples were obtained from the ELK Berlin Chest Hospital (Berlin, Germany). Histological data (histological subtype and grade) can be found in Additional File 1. Appropriate consent in line with institutional requirements was obtained from all patients. The study protocol was approved by the local ethics committees. All samples were fixed with RNAlater and stored immediately at -80°C.

### RNA Preparation

RNA was extracted using the RNeasy Mini Kit (Qiagen, Hilden, Germany), following the manufacturer's instructions. DNA contamination was eliminated by employing the RNase-Free DNase Set (Qiagen, Hilden, Germany). And RNA was reversely transcribed into cDNA using the Superscript First Strand Synthesis System for RT-PCR (Invitrogen, Carlsbad, CA). Nucleotide contents were measured on a Nanodrop^® ^ND-1000 spectral photometer (Nanodrop Technologies, DE, USA).

### DNA Extraction

Washed research sperm (NW Andrology & Cryobank Inc., Spokane, WA, USA) was lysed as previously described [24]. Tumor tissues were lysed by adding 100 μl lysis buffer (50 mM Tris pH 8.4, 1 mM EDTA, 0.5% [v/v] Tween^®^20) and proteinase K (20 mg/ml, Carl Roth, Karlsruhe, Germany) and incubated for 8 h at 60°C and 1000 rpm in a thermomixer. Another 10 μl proteinase K was added and the samples were incubated for another 4 h. Eighty μl of the lysate was subjected to the bisulfite conversion reaction as described below. Genomic DNA from the lysate was extracted as follows: Twenty μl tumor tissue lysate was mixed with 180 μl water, 250 μl binding buffer (6 M guanidiniumthiocyanate, 0.1 M Tris; pH 7.5) and 250 μl ethanol (molecular biology grade, ≥ 99,8%). The mixture was transferred onto a NucleoSpin Extract II Column (Macherey & Nagel, Dueren, Germany) and was centrifuged at 14,000 × g for 3 min, and the flow-through was discarded. The immobilized DNA was washed with 700 μl wash buffer (150 mM Tris pH 7.4, 85% ethanol) followed by three rounds of centrifugation (14,000 × g, 1 min). The spin column was incubated for 10 min at 60°C with open lids in a thermomixer in order to evaporate residual ethanol. Forty μl water was added to the centre of the membrane and incubated for 1 min. The DNA was eluted by centrifugation for 1 min at 14,000 × g.

DNA concentration was quantified using a Nanodrop^® ^ND-1000 spectral photometer (Nanodrop Technologies, DE, USA).

### Bisulfite Conversion

Lysates from tumor tissues, universally methylated DNA (CpGenome™ Universal Methylated DNA, Millipore, MA, USA), and extracted DNA from sperm were filled up with lysis buffer to a final volume of 80 μl and treated as follows: Eighty μl bisulfite reagent (65% ABS, pH 5.3 [TIB Chemicals, Mannheim, Germany]) and 40 μl denaturation reagent (0.07 g/ml Trolox [Sigma-Aldrich, St. Louis, MO] in THFA [VWR International, Darmstadt, Germany]) were added and the mixture was incubated for 45 min at 85°C and 1,000 rpm in a thermomixer. Two hundred and fifty μl ethanol and 250 μl binding buffer were added and the bisulfite converted DNA was extracted as described above with the following modification: washing was carried out with 700 μl desulphonation buffer (250 mM NaOH, 75% ethanol) after the first washing step.

DNA concentration was quantified via UV spectrophotometry as described above.

Bisulfite converted sperm DNA (known to be unmethylated at many loci [25]) and converted universally methylated DNA were mixed to produce samples with a defined percentage of methylation.

### SHOX2 DNA Methylation Real-time PCR Assays

Relative DNA methylation of the *SHOX2 *locus compared to total DNA (determined via ACTB reference in a duplex PCR reaction) was quantified using the Epi *pro*Lung BL real-time PCR kit (Epigenomics AG, Berlin, Germany) following the instructions for use, but only 0.25 μl DNA were subjected to PCR.

Relative DNA methylation of the *SHOX2 *locus referred to total DNA as determined with a methylation-unspecific *SHOX2 *assay was analyzed in 20 μl duplex PCR reaction with the following composition: Thirty-five mM Tris, pH 8.4, 6 mM MgCl_2_, 50 mM KCl, 5% glycerol, 0.25 mM each dNTP, 3 U FastStart Taq DNA polymerase [GMP grade, Roche Diagnostics, Mannheim, Germany], primers (0.3 μM 5'-ggttttgagtaattaatagaaat-3', 0.3 μM 5'-ctctttctattctctcttc-3', 0.8 μM 5'-gttttttggatagttaggtaat-3', 0.8 μM 5'-cctcctaccttctaaccc-3'), 1.5 μM each blocker (5'-taatttttgttttgtttgtttgattggggttgtatga-SpacerC3-3', 5'-acccaacttaaacaacaaacccttta-SpacerC3-3'), 0.6 μM each detection probe (5'-6-R6G-tgaatttgttgatttttgtgggt-BHQ1-3', 5'-6-FAM-ctcgtacgaccccgatcg-BBQ-650-3') and 20 ng DNA (according to UV quantitation). The same PCR cycling was used as described in the instructions for use of the Epi *pro*Lung BL real-time PCR kit.

For each sample and each of the two assays as described above, the relative methylation values were determined using the ΔΔCT method [26,27]. Bisulfite converted universally methylated DNA (as described above) was used as reference DNA to transform ΔCT into ΔΔCT. Therefore, a ΔΔCT_Sample _= 0 refers to 100% methylated DNA.

### SHOX2 Copy Number Real-time PCR Assay

The methylation-unspecific quantification of the total amount of bisulfite converted *SHOX2 *copies was performed in 20 μl PCR reaction with the following composition: Tris, pH, MgCl_2_, KCl, glycerol, dNTPs, FastStart Taq DNA polymerase as described above, 0.75 μM each primer (forward: 5'-ggttttgagtaattaatagaaat-3', reverse: 5'-ctctttctattctctcttc-3'), 0.3 μM detection probe (5'-6-FAM-tgaatttgttgatttttgtgggt-BHQ1-3') and 20 ng DNA (according to UV quantitation). Ten ng of bisulfite converted DNAs (0%, 25%, 50%, 75%, and 100% methylated) were used as standards to convert the CT of a given sample into ng of DNA. Each methylation mixture and each sample were measured in triplicates and the mean was calculated.

PCR was performed using a 7500 Fast Real-Time PCR machine (Applied Biosystems, CA, USA) using the following temperature profile: 15 min at 95°C of initial denaturation followed by 45 cycles with 15s, 95°C and 30s, 58°C.

### SHOX2 qRT PCR Assay

qPCR was carried out in 20 μl reaction with the composition as described above but with the following oligonucleotides:

*SHOX2 *variants *a *and *b*: forward: 5'-gtgttctcataggggccgccagc-3', reverse: 5'-acagcgctgtccagctgcagctgcg, probe: 5'-FAM-tcgcaccttatgtcaacgtaggtgc-BHQ1-3'

SHOX2a (NM_006884): forward primer and probe as depicted for both variants, reverse: 5'-ggcgtcacgttgcaatgactat-3'

SHOX2b (NM_003030): forward primer and probe as depicted for both variants, reverse: 5'-cagctgcgcctgaacctgc-3'

HPRT1 (NM_000194): forward: 5'-tgatagatccattcctatgactgtaga-3', reverse: aagacattctttccagttaaagttgag-3', probe: 5'-FAM-cccctgttgactggtcattacaatagctc-BHQ1-3'

SDHA (NM_004168): forward: 5'-tgggaacaagagggcatctg-3', reverse: 5'-ccaccactgcatcaaattcatg, probe: 5'-FAM-ccatttctgctcagtatccagtagtgg-BHQ1-3'

PCRs were performed using a 7500 Fast Real-Time PCR machine (Applied Biosystems, CA, USA) with the following temperature profile: 30 min/25°C, 15 min/95°C and 40 cycles with 15s/95°C and 1 min/60°C.

### Array CGH

Whole-genome array comparative genomic hybridisation (CGH) analysis was performed using a 1M oligonucleotide array (Agilent, Santa Clara, CA) according to protocols provided by the manufacturer. Image analysis, normalization and annotation were based on Feature Extraction 9.1 (Agilent, Santa Clara, CA) using the default settings, and visualization of data was performed with the DNA Analytics software (Agilent, Santa Clara, CA). DNA from the matched NAT tissue was used as a reference sample for the respective tumor DNA sample.

### Statistical Analysis

The Kendall's tau and Spearman rank correlation were used to test whether DNA methylation and gene amplification were statistically dependent. One-sided p-values were reported. The Wilcoxon Rank-Sum was used to test wether SHOX2 DNA methylation, gene amplification and RNA expression differed between the histological tumor subtypes.

## Results

**Figure 1:**
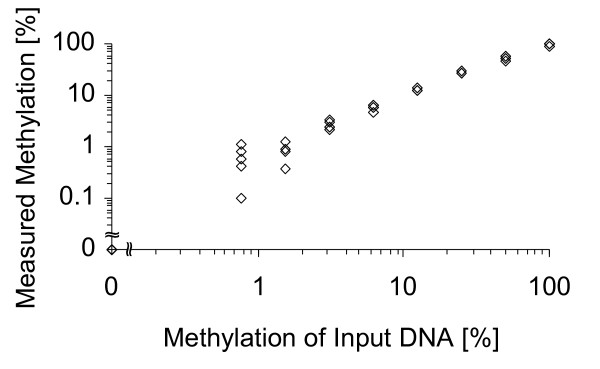
**Response curve for the quantification of *SHOX2 *DNA methylation**. Mixtures of bisulfite converted DNA from sperm and bisulfite converted artificially methylated DNA were used as template DNA. Each methylation mixture (0, 0.8, 1.6, 3.1, 6.2, 12.5, 25, 50, and 100%) was measured in five replicates.

*SHOX2 *DNA methylation has previously been reported to be applicable for the diagnosis of lung cancer based on the analysis of bronchial lavage samples [14]. In this study, the *SHOX2 *DNA methylation was quantified in tumor tissues and morphologically normal adjacent tissues from 55 lung cancer patients. In brief, this assay represented a duplex real-time PCR consisting of a HeavyMethyl (HM) assay [28] for sensitive and quantitative detection of *SHOX2 *DNA methylation (promoter region of transcript variant b [14]) and a reference PCR for quantification of the total DNA using the *ACTB *locus. A response curve of this assay is given in figure 1 with different mixtures of bisulfite converted DNA from sperm (known to be unmethylated at many loci [25]) and bisulfite converted DNA from artificially methylated DNA. The assay allowed for highly accurate quantification of the *SHOX2 *DNA methylation over a broad range of relative methylation of the template DNA.

**Figure 2:**
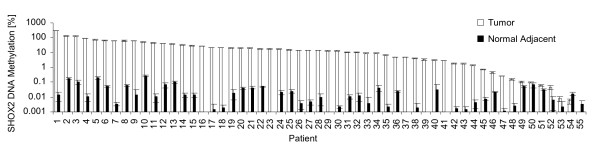
***SHOX2 *DNA methylation in tumor tissue and morphologically normal adjacent tissue (NAT) from 55 lung cancer patients**. Ninety-six percent (53/55) of patients exhibited a higher methylation value in tumor tissue as compared to the matched NAT. Fifty-five (30/55) of the NAT showed a low *SHOX2 *methylation of 0.01 to 0.3%. *SHOX2 *DNA methylation levels in three tumors were significantly higher than 100%.

As indicated in figure 2, *SHOX2 *was methylated in the vast majority of tumors. Ninety-six per cent (53 out of 55) of matched pairs showed a higher methylation level in tumor tissues as compared to the matched NAT from the same patient. The analyzed SHOX2 was significantly higher methylated in squamous cell carcinomas as compared to adenocarcinomas (p = 0,0006 [Wilcoxon Rank-Sum test], Additional File 1). Low *SHOX2 *DNA methylation levels were also present in 55% (30 out of 55) of the NAT ranging from 0.01% to 0.3%. Interestingly, the *SHOX2 *methylation level of three patients' DNAs was above the theoretically expected maximum of 100% (patient #1: 291%, patient #2: 130%, patient #3: 122%). This observation is unlikely to be a measurement artefact since the response curve in figure 1 showed high accuracy of the detection assay.

**Figure 3:**
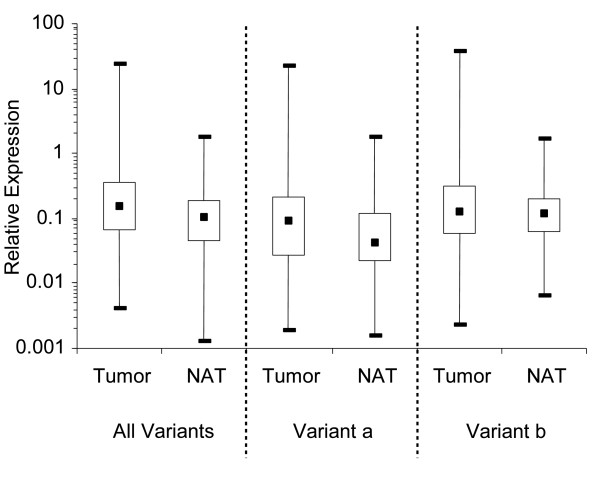
***SHOX2 *RNA expression in tumor tissue and morphologically normal adjacent tissue (NAT) from lung cancer patients**. Three qRT-PCR assays specific for both variants and variant a (NM_006884) and b (NM_003030), respectively, were used. Measurements were carried out in triplicates and normalized to *SDHA *and *HPRT1 *genes. Valid results were obtained for NAT and tumor tissues from 51 lung cancer patients.

DNA methylation within the promoter region of a gene is known to be part of a regulatory mechanism that is able to silence gene expression [for review: [29]]. The correlation between *SHOX2 *DNA methylation and gene expression was analyzed with three different quantitative real-time PCR assays. Two assays that were specific for the transcript variants *SHOX2*a and *SHOX2*b, respectively, which are suggested to be transcribed from alternative promoters [15] were designed. The third assay was designed to detect the total amount of *SHOX2 *RNA transcripts. The relative expression level of the two *SHOX2a *and *b *variants alone, as well as the total amount of *SHOX2 *RNA in tumor tissues and normal tissues, is shown in figure 3 and Additional File 1. A downregulation of *SHOX2 *expression due to a hypermethylation of the *SHOX2 *region in tumor tissues could not be observed. Contrarily, the *SHOX2 *expression seemed slightly elevated in tumor tissues, however, this was not statistically significant.

**Figure 4:**
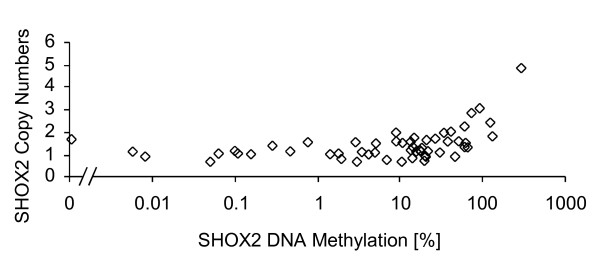
**Correlation of gene amplification and DNA methylation of the *SHOX2 *locus**. *SHOX2 *copy numbers were determined by relating the total amount of *SHOX2 *copies (determined with real-time PCR) to the amount of total DNA as quantified by UV. Methylation of the *SHOX2 *locus was measured using a duplex PCR consisting of a HM assay for sensitive quantification of methylated *SHOX2 *copies and an *ACTB *reference assay. Means of a triplicate measurement are shown. Gene amplification and DNA methylation correlate highly (p = 0.0002).

Genomic gain on chromosome 3q, where *SHOX2 *is located, has been recognized as one of the most prevalent and significant alterations in lung cancer [19-23]. Therefore, the *SHOX2 *copy numbers were analyzed in order to investigate a potential correlation of *SHOX2 *DNA methylation and gene amplification. Quantities of *SHOX2 *copy numbers were characterized by the ratio of total *SHOX2 *DNA (to the total amount of DNA). The amount of total DNA was quantified by UV spectrophotometry. Quantities of *SHOX2 *copies were measured using a real-time PCR assay which specifically amplifies bisulfite converted copies of the *SHOX2 *gene (methylated and unmethylated). Even though the primers used for this assay did not include CpG islands, a PCR bias (i.e. preferred amplification of unmethylated or methylated DNA), had to be excluded [30-32]. Otherwise, a more efficient amplification of methylated DNA would result in overestimated *SHOX2 *copy numbers in methylated specimens. Therefore, mixtures (0 to 100%) of bisulfite converted DNA from sperm and from artificially methylated DNA were analyzed with respect to a potential PCR bias. 20 ng DNA (according to UV) was subjected to a triplicate real-time PCR analysis. The resulting CT were 28.70 ± 0.33 (0% mixture), 28.55 ± 0.13 (25%), 28.54 ± 0.30 (50%), 28.37 ± 0.12 (75%), and 28.16 ± 0.11 (100%), respectively, indicating that no significant PCR bias exists for this assay. The determined *SHOX2 *copy numbers in relation to DNA methylation in the 55 tumor tissue samples is shown in figure 4 and Additional File 1. High *SHOX2 *DNA methylation was found to correlate strongly with gene amplification (p = 0.0002 [Kendall's tau rank correlation], p < 0.0001 [Spearman rank correlation]). Amplification of the *SHOX2 *gene locus did not differ significantly between adeno- and squamous cell carcinomas.

**Figure 5:**
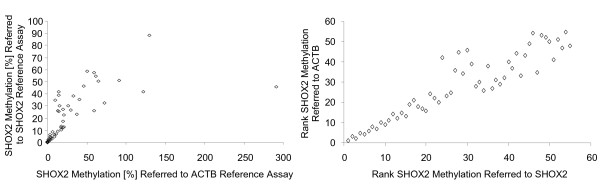
**Correlation of *SHOX2 *methylation in 55 tumors as determined using *ACTB *and *SHOX2 *as references**. Left: Scatter plot of *SHOX2 *methylation. *SHOX2 *methylation was determined with an *ACTB *reference assay (x-axis) and a methylation unspecific *SHOX2 *reference assay (y-axis), respectively, for quantification of total DNA in a duplex PCR. Means of a triplicate measurement are shown. Right: Scatter plot of ranks. The methylation values as determined with both reference assays were ranked and the respective ranks were correlated.

DNA methylation of the *SHOX2 *locus as shown in figure 4 was determined using a *SHOX2*/*ACTB *duplex real-time PCR. The *SHOX2 *assay specifically amplified methylated *SHOX2 *gene copies, while the *ACTB *primers amplified methylated and unmethylated copies of the *ACTB *gene. Thus, it cannot be excluded that the apparent higher relative *SHOX2 *methylation in tumors with *SHOX2 *gene amplification is simply due to the presence of higher numbers of total *SHOX2 *copies and therefore also a higher number of methylated copies. This would result in an overestimation of the relative methylation in samples with amplification of the *SHOX2 *locus. A sample where both *SHOX2 *gene copies are methylated would result in a methylation level of 100%. However, a methylation level of 200% would indicate that the *SHOX2 *locus is duplicated when a different locus, i.e. *ACTB*, is used for quantification of the total DNA. Thus, the apparent relative methylation of the *SHOX2 *locus correlates with the copy numbers due to the usage of a different locus as a reference assay. To circumvent this overestimation of relative *SHOX2 *methylation a reference assay which is located at the same locus was applied, where completely methylated *SHOX2 *gene is determined as 100%, independent of agene amplification. Thus, a duplex PCR was developed, where methylated *SHOX2 *copies were directly correlated to the total copy number of the *SHOX2 *gene itself. The correlation of the *SHOX2 *methylation as determined with an *ACTB *and a *SHOX2 *assay for quantifying total DNA is shown in figure 5 and Additional File 1. Using this duplex assay, none of the samples was determined as methylated above 100%. This confirmed that methylation determined by the *ACTB *reference assay was overestimated for samples showing gene amplification. However, the DNA methylation levels quantified by both reference assays showed strong correlation (p < 0.0001 [Kendall's tau and Spearman rank correlation]). In addition, DNA methylation quantified with the *SHOX2 *reference assay still showed a significant correlation with gene amplification (p = 0.002 [Kendall's tau and Spearman rank correlation]).

**Figure 6:**
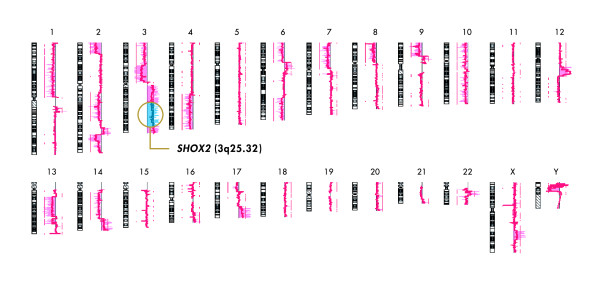
**Summary of comparative genomic hybridization.** Abnormalities identified in tumor tissue from patient #1 as compared to the corresponding NAT. Chromosomal amplification is clearly demonstrated for 3q comprising the *SHOX2 *locus.

Tumor tissue derived from patient #1 showed a very high methylation rate of 291% (referred to *ACTB*) and 46% (referred to total *SHOX2 *copies), respectively, as well as a strong copy number amplification of the *SHOX2 *gene (4.9 fold). This tumor DNA was analyzed in more detail using array CGH technology. A summary of the CGH results are depicted in figure 6 showing that the complete q arm of chromosome 3 is overrepresented in the tumor. Taken together, this data clearly demonstrate that the *SHOX2 *locus is highly amplified in the tissue derived from patient #1. In summary, the presented results indicate that DNA methylation of the *SHOX2 *gene highly correlates with the amplification of its chromosomal location.

## Discussion

*SHOX2 *DNA methylation, operating as a biomarker at a high specificity and sensitivity in a group of lung cancer patients and critical controls based on the analysis of bronchial lavage samples, has recently been proven to be a suitable means for diagnosing lung cancer [14]. The biomarker also worked with samples that contained no visible tumor cell content. Since it has also been found that genomic gain at chromosome 3q, including *SHOX2*, is one of the most prevalent and significant alterations in lung cancer [19-23], the objective of this study was to investigate the relationship between gene amplification, expression and DNA methylation of *SHOX2*. In this study, it was shown that in 96% of all cases of lung tumor patients, the *SHOX2 *gene is hypermethylated and frequently accompanied by increased copy numbers of the respective locus. Hence, the positive performance of the *SHOX2 *DNA methylation as a biomarker in cytologically negative bronchial lavage samples might be due to a correlation of locus amplification and DNA methylation in tumor cells. Genomic amplification did not lead to a statistically significant higher expression of *SHOX2 a *or *b *transcripts as compared to the respective NAT. However, it has to be considered that *SHOX2 *mRNA levels were measured in homogenized (lysed) tumor tissues. Therefore, the extracted mRNA from tumor tissues is susceptible to contamination by non-neoplastic cells. This contamination might mask tumor-specific alterations. Such contamination affects mRNA expression analyses much more dramatically as compared to DNA methylation and gene amplification since the expected ranges (fold changes) of mRNA expression between different cell types can be much higher. In cancer research, the development of laser microdissection (LMD) systems has addressed this dilemma and could be implemented in the future to study *SHOX2 *expression in distinct tumor cell types.

Interestingly, NAT also showed low levels of methylation (< 0.3%). This observation might arise from a potential field effect, leading to alterations of methylation in normal components adjacent to the tumor, or from the existence of a subpopulation of cells with similarities to the tumor cells on an epigenetic level. Alterations of methylation in NAT and the existence of a subpopulation of cells within the normal epithelia with epigenetic similarities to the tumor cells have previously been reported in several cancers [33,34]. In addition, tumor development and progression occur as an interaction between tumor cells and their stromal environment [for review: [35,36]], and the concept of epithelial to mesenchymal transition (EMT) as a driver of tumor progression has become more important in recent years [37,38]. Thus, cells, which might not allow for a morphological cancer diagnosis do already harbour the cancerogenic information by their epigenetic state. However, in order to address the question of whether methylation in NAT comes from such a field effect or is also present in tissue from healthy individuals, normal components at larger distances from the tumor or in tissue from healthy controls would have to be analyzed.

Both, a potential field effect on an epigenetic level, as well as a concerted mechanism of gene amplification and DNA methylation, renders DNA methylation an attractive source of biomarkers for the diagnosis of cancer in body fluids where sensitive detection is required. The reinforced epigenetic signal, together with chromosomal rearrangements and thus locus amplifications, may be part of a cellular mechanism during tumorigenesis. Therefore, DNA methylation may be preferrentially employed as a diagnostic test compared to methods based on cell counting. Additionally, the detection and accurate quantification of DNA is easier as compared to protein and RNA, due to the higher stability of DNA in quantitative real-time PCR. This study also indicates that a combination of genome-wide DNA methylation analysis, i.e. MeDIP and DMH, and CGH is effective for the screening of candidate marker genes and therefore might also influence the discovery of novel biomarkers for clinical applications. A multiplexing of such markers, ideally from different chromosomes might further improve a diagnostic test.

However, the functional relationship between DNA methylation and amplification as well as the underlying pathomechanism remains to be elucidated. It is not yet clear whether DNA methylation is a response reaction to locus amplification or if DNA hypermethylation promotes destabilization of the genome and with that gene amplification. However, it is well acknowledged that a genome-wide hypomethylation induces chromosomal instability [for review: [11]] and that genome-wide hypomethylation in cancer goes hand-in-hand with gene-specific hypermethylation, e.g. of tumor suppressor genes [for review: [39]]. Thus, *SHOX2 *hypermethylation in lung cancer might be indicative of an overall hypomethylation of the corresponding chromosomal region and therefore of genetic instability which results in gene amplification.

## Conclusions

The tumor-specific hypermethylation of *SHOX2 *and its frequent gene amplification in lung cancer renders *SHOX2 *DNA methylation is an attractive biomarker for lung cancer when sensitive detection is desired, i.e. in bronchial lavage specimens.

## Abbreviations

ABS: Ammonium Bisulfite; BL: Bronchial Lavage; CGH: Comparative Genomic Hybridization; CT:Cycle Treshold; DMH: Differential Methylation Hybridization; EDTA: Ethylenediaminetetraacetic Acid; HM: HeavyMethyl; MeDIP: Methylated DNA Immunoprecipitation; NAT: Normal Adjacent Tissues; *SHOX2*: Short Stature Homeobox 2; THFA: Tetrahydrofurfuryl Alcohol

## Competing interests

The authors DD, VL, CK, and AS, are or have been employees and/or shareholders of Epigenomics AG (Berlin, Germany), a company that aims to commercialize DNA methylation markers. The other authors report to have no conflict of interest regarding the topic of the article.

## Authors' contributions

MF, DD, and KS drafted the manuscript, conceived and coordinated the study. BS, VL, CK, and GR participated in the design of the study and its supervision and revised the manuscript. GL, SW, FS and JM provided and characterized the sample material. AS performed the real-time PCR experiments. FE conducted the array CGH experiments and analysis. All authors read and approved the final version of the manuscript.

## Pre-publication history

The pre-publication history for this paper can be accessed here:

http://www.biomedcentral.com/1471-2407/11/102/prepub

## Supplementary Material

Additional file 1 **Patient information and *SHOX2 *data**. This excel spreadsheet (.xls) contains the histological subtypes and the measured SHOX2 methylation, gene amplification and expression data for each patient. Click here for file
